# Change deafness for real spatialized environmental scenes

**DOI:** 10.1186/s41235-017-0066-3

**Published:** 2017-06-28

**Authors:** Jeremy Gaston, Kelly Dickerson, Daniel Hipp, Peter Gerhardstein

**Affiliations:** Army Research Laboratory, Human Research and Engineering Directorate, Adelphi, MD USA

**Keywords:** Change deafness, Spatial audio, Similarity effects, Cued-recall, Environmental sound

## Abstract

The everyday auditory environment is complex and dynamic; often, multiple sounds co-occur and compete for a listener’s cognitive resources. ‘Change deafness’, framed as the auditory analog to the well-documented phenomenon of ‘change blindness’, describes the finding that changes presented within complex environments are often missed. The present study examines a number of stimulus factors that may influence change deafness under real-world listening conditions. Specifically, an AX (same-different) discrimination task was used to examine the effects of both spatial separation over a loudspeaker array and the type of change (sound source additions and removals) on discrimination of changes embedded in complex backgrounds. Results using signal detection theory and accuracy analyses indicated that, under most conditions, errors were significantly reduced for spatially distributed relative to non-spatial scenes. A second goal of the present study was to evaluate a possible link between memory for scene contents and change discrimination. Memory was evaluated by presenting a cued recall test following each trial of the discrimination task. Results using signal detection theory and accuracy analyses indicated that recall ability was similar in terms of accuracy, but there were reductions in sensitivity compared to previous reports. Finally, the present study used a large and representative sample of outdoor, urban, and environmental sounds, presented in unique combinations of nearly 1000 trials per participant. This enabled the exploration of the relationship between change perception and the perceptual similarity between change targets and background scene sounds. These (post hoc) analyses suggest both a categorical and a stimulus-level relationship between scene similarity and the magnitude of change errors.

## Significance

Our laboratory, in addition to basic applied research, works closely with the test and evaluation community to quantify the safety and effectiveness of communication devices and hearing protection systems and other forms of personal protective equipment. An important part of this interaction is supporting the development of test requirements and methodologies that enable systematic evaluations of systems under conditions that mirror those of the real world. However, the reality of tests and evaluations is that they are based on simple, well-understood behaviors that are limited in scope. This is generally advantageous because the goal of such tests is to produce reliable and valid results that are highly reproducible across a variety of laboratories. For example, typical measures include speech intelligibility with high noise, single-source sound localization, and auditory detection. These measures represent worst-case conditions for simple perceptual behaviors, but fail to account for the kind of challenges faced during perception under real world complexity and variability. The current study presents research that was inspired by our interactions with the test and evaluation community and the realization that there is a significant gap in the understanding of how human performance will be affected in real-world complex environments. The change deafness phenomenon provides a balance between the traditional psychophysical approach and real-world complexity while enabling systematic characterizations of performance. The research presented here can inform the development of future test and evaluation requirements and procedures.

## Background

An ‘auditory scene’ is broadly defined as an array of concurrent sound sources (Gygi & Shafiro, 2010). Auditory scenes can be as simple as a set of pure tones, one or more chords, or a complex array of environmental sounds, like that encountered on a busy city street corner, in a restaurant kitchen during the lunch rush, or in a stadium filled for a rock concert. Increasing the complexity of an auditory scene increases the perceptual and cognitive demands for processing and can lead to misperceptions. Such misperceptions can manifest as inaccurate or inaccessible perceptual representations (Darwin et al. 1972; Näätänen & Winkler, 1999), which can be influenced by top-down factors such as attention, attributions of relevance, and interactions between short- and long-term memory processes (e.g., Zimmermann et al., 2016; Kidd et al., 2008; Gregg & Samuel, 2008; Cowan, 2001). Informational factors, such as stimulus similarity and uncertainty, also contribute to inaccurate or inaccessible perceptual experiences (Dickerson & Gaston, 2014 for review).

An example of the pervasiveness of perceptual errors is evident in the ‘change deafness’ phenomenon, which describes the failure of listeners to notice changes when they are embedded within complex auditory scenes. Change deafness, like a similar finding in the visual literature, change blindness, demonstrates that, despite a subjective impression of coherence and completeness, perceptual experience is incomplete and can be inaccurate. Change deafness has been demonstrated across several auditory domains to include speech (Sinnett et al. 2006; Vitevitch, 2003), environmental sounds (Gregg & Samuel, 2008, 2009; Eramudugolla et al. 2005; Gregg & Snyder, 2012), and music (Agres & Krumhansl, 2008). Several authors have also demonstrated the difficulty of auditory change perception tasks using artificial (synthesized) scenes such as pure tones shaped into scenes via amplitude modulation and shaped noise arrays (e.g., Constantino et al., 2012). A review of the recent change deafness literature (Dickerson & Gaston, 2014) noted that changes are missed 20–50% of the time depending on various perceptual and cognitive factors. For example, similarity between the sound that is changed and the other sounds in the scene influences change perception accuracy, with changes that are acoustically and semantically dissimilar from background sounds producing fewer errors (Gregg & Samuel, 2009), as would be expected from a signal-noise ratio perspective. Gregg et al. (2014) demonstrated that familiarity is also an important factor in driving change perception errors by showing that temporally scrambled and unrecognizable sounds produced significantly more errors than unscrambled and recognizable sounds. The manner in which a change occurs also appears to influence performance. Constantino et al. (2012) found that listeners performed better when a new sound was added to a scene than when a sound was deleted. Finally, change deafness seems to be influenced by attention, namely cueing or directing attention to the spatial location of a changed sound source can reduce the frequency of change perception errors (Eramudugolla et al., 2005; Backer & Alain, 2012).

The reportedly positive effect of providing a cue to the location of a change suggests that spatial position and spatial separation may be useful for perceptually segmenting a scene, which in turn may reduce change perception errors. Studies addressing the relationship between spatial separation and change perception, however, are few, limited to virtual audio manipulations, and are generally not in agreement. Gregg and Samuel (2008) found no segregation advantage for spatially separated sources in a virtual array, whereas Eramudugolla et al. (2005) found that spatial separation resulted in significantly fewer change perception errors. More generally, spatial position can be a cue to successful perceptual segregation (e.g., Bregman, 1993; Yost, 1993, 1997) and a number of psychophysical studies have shown (auditory) spatial cues to provide beneficial effects for perceptual performance (e.g., Broadbent, 1954; Best et al., 2006; Jones & Litovsky, 2011), including auditory search (Eramudugolla et al., 2008). Specifically, spatial separation has been shown to provide a reduction or elimination of informational masking effects (e.g., Ihlefeld & Shinn-Cunningham, 2008; Kidd et al., 1994), a phenomenon that may share common perceptual mechanisms with patterns in reports of change deafness (Dickerson & Gaston, 2014). There is clearly reason to expect that spatial separation may reduce change perception errors by reducing perceptual ambiguity, but as was previously mentioned, the literature on this topic as it relates to change deafness is sparse and conflicting. The present study uses a physical multi-speaker array and compares scenes with spatially separated versus spatially co-located sounds to systematically evaluate the role of spatial separation in modulating change errors. The use of sounds presented over speakers in the free-field, rather than the use of a virtual spatial manipulation, is an important methodological change from previous studies, as it is often the case that virtual spatial arrays are more often lateralized than truly localized (Yost, 1993) and artifacts associated with headphone lateralization, or the use of a generic head-related transfer function could, in part, explain the mixed results of previous studies investigating the role of spatial cues in change deafness.

In addition to the spatial manipulation, we follow the path of others in this area by examining how the type of change influences perceptual errors. In the current study, we instantiate changes via source additions and source removals. Change deafness studies in the past have manifested changes via source removals (e.g., Eramudugolla et al., 2005), as in ‘token and type changes’ (Gregg and Samuel, 2009), where a source in the scene is replaced with a signal that is either semantically and acoustically dissimilar (token change) or only acoustically dissimilar (type change), a ‘switch’ in which a sound is replaced with a different sound (Gregg et al., 2014), or a position ‘swap’, where two sounds change spatial position (Backer & Alain, 2012). Only Constantino et al. (2012) appear to have looked at both the addition and the deletion of a source within a single study context. Constantino et al. (2012) found that the addition of a source was easier to detect because the new source ‘pops-out’ from the background, compared to a deletion, in which the information in each frequency band must be iteratively compared. The present study examines performance for both additions and removals, as there is some suggestion (from Constantino et al., 2012, and others) that fewer errors should occur for source additions, as these changes will be perceived as ‘onset events’ and may pop out. Onset events are likely to elicit an automatic allocation of attention (e.g., Samuel & Weiner, 2001), which is known to facilitate change perception in both vision (Miller, 1989) and audition (Sussman et al., 2003). Thus, the addition of a new sound to the scene should be especially salient, causing participants to make fewer errors in the addition than in the removal condition. Although source additions may be more salient events, all of the scenes in the present study commence with the same number of sounds. Thus, an addition will result in a scene that has two more sources than in the sound removal condition. If there is some limit on the number of stimuli that can be represented in memory, then there should be more errors in the source addition condition with a trend toward reductions in errors as the size of scene two decreases (from 5 or 4, to 3 in the Addition, No-change, and Removal conditions, respectively).

This idea that the scene size, and therefore memory load, plays a role in change perception errors has been examined previously. Gregg and Samuel (2008) found evidence for change deafness (high errors) despite generally accurate performance on a cued recall task. In vision, Mitroff et al. (2004) report a similar finding; however, their results are less clear. Mitroff et al. (2004) in fact reported that memory for pre- and post-change scenes was preserved even when participants reported no awareness of a change, but in follow-up experiments they found that the stored representations are fragile; simply reversing the question order from cued-recall first to cued-recall last lead to significant decrements in recall. To further explore the interaction between recall accuracy and change perception errors and assess memory for scene elements, the present study presents participants with a cued recall task following each change perception trial.

Finally, change deafness is usually characterized in terms of hits, or accuracy, in indicating that a change has occurred. Change deafness, the failure to notice a change that has occurred, would be most directly measured by looking at hits or misses (e.g., Gregg & Samuel, 2008). However, restricting analyses to accuracy is a potential limitation because hit rates can be substantially influenced by listener response biases. In Signal Detection Theory (SDT) (Macmillan & Creelman, 2005), changes in response bias correspond to changes in decision criteria that ultimately result in systematic changes in hit and false alarm rates. These systematic changes can be modeled in receiver operator characteristic space and show that, across changes in criteria, sensitivity remains the same. In the change blindness literature, the influence of response bias is recognized and thus analyses typically report performance based on SDT measures of sensitivity in addition to accuracy measures (e.g., Mitroff et al., 2004). Although SDT approaches have not been broadly applied in the change deafness literature, there are notable examples (e.g., Eramudugolla et al., 2005; Gregg & Samuel, 2008; McAnally et al., 2010; Puschmann et al., 2013a, 2013b) that report evidence of change deafness despite using a bias-free measure of sensitivity. Here, we report measures of accuracy and SDT measures to examine patterns of hits and false alarms as well as a bias-free measure of sensitivity (*d’*) using an AX (same-different) task. We refer to the phenomenon of ‘change deafness’, but will also use the term ‘change discrimination’ where appropriate to denote the experimental procedure underlying measurement of the phenomenon.

To summarize, the present study fills a gap in the emerging change deafness literature by manipulating several common factors thought to influence change perception performance. We address the mixed results over the role of spatial cues in change deafness by comparing performance for spatially distributed or spatially co-located scenes using real spatial sources over a loudspeaker array. We examine two common change implementation strategies to investigate both the possibility that pop out could occur for additions and the secondary goal of evaluating set size effects. Finally, we present accuracy and SDT analyses together to eliminate the possibility that change discrimination errors are not simply an artifact of listener bias.^1^

## Methods

### Participants

Twenty-six adults were recruited from a temporary employment agency and paid for their participation. All participants had normal hearing, measured as a threshold at or below 25 dB (HL) for octave frequencies between 500 and 8000 Hz. Three additional participants completed the study, but their data were excluded from the analysis; two were identified as outliers, their overall performance was greater than 2 SE above the group mean, and one who produced a pattern of responding completely different from the rest of the sample. The voluntary, fully informed consent of the persons participating in this research was obtained as required by U.S. Army human use regulations (U.S. Department of Defense, 1999; U.S. Department of the Army, 1990). Thirteen participants completed the multi-speaker condition and the remaining 13 participants completed the mono-speaker condition, with condition assignment randomized across participants.

### Stimuli

Stimuli consisted of 25 sounds selected to be representative of a typical outdoor urban or suburban environment (see Table 1 for the sound list). The majority of sounds used in this study were downloaded from the online database *freesound* (freesound.org), and others were taken from a separate internal database. Speech and music were specifically excluded, as such stimuli can be quite distinct from environmental sounds, and can be difficult to present at short durations while maintaining fidelity and a naturalistic percept. In addition, this set of sounds was characterized in a pilot study using internal personnel, with each of the 25 sounds exceeding 80% identification accuracy when presented in isolation.

**Table 1:** Each of the listed 25 sounds represents common outdoor environmental sounds. Each of these sounds served as a target on 15 Add and 15 Remove trials

Stimulus list				
Bicycle bell	Cell phone ringing	Helicopter hovering	Jingle bell	Tank passing by
Bicycle chain	Cicadas	Helicopter passing by	Motorcycle accelerating	Truck idling
Bicycle chain and flywheel	Crickets	Jackhammer	Prop plane	Truck accelerating
Bus air brake	Dog barking	Jet plane	Pouring water	Turning on shopvac
Bus idling	Dog shaking head	Jet passing by	Shopvac running	Footsteps

Sound sources included in the current study were truncated to 1 s samples when the original file was longer in duration. Care was taken, however, to preserve onset and offset information. To truncate selected samples, redundant or repeating segments were removed and silent intervals occurring at the beginning or end of the file were shortened or removed. In an effort to minimize listener use of potential loudness cues across trials, three versions of each stimulus were recorded, namely ‘loud’, ‘neutral’, and ‘quiet’, stimuli were generated by averaging the root mean square (RMS) amplitude of all of the stimuli and creating alternative versions that were +3 dB RMS, 0 dB RMS, and –3 dB RMS relative to the average across all stimuli. Thus, on any given change trial the change could be louder or quieter than the contextual elements, independent of whether the change was the addition or removal of a sound.

### Apparatus

**Fig. 1:**
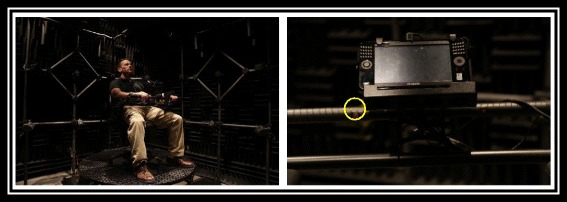
*Left* panel depicts a listener seated in the center of the speaker array in the sphere room at the Environment for Auditory Research. The elevated platform allows for the listener to be centered within a ring of speakers positioned every 22.5°. The panel on the *right* depicts the micro PC used to present trial information to the participant. Participants responded using the* red buttons* mounted to the handle bar (positioned to the immediate *left* and *right* of the micro PC, highlighted in the *yellow circles* in the image at *right*)

All participants completed the experiment in the Sphere room of the Army Research Laboratory’s Environment for Auditory Research facility (for detailed specifications see: Henry et al., 2009). The Sphere room is a semi-anechoic space containing a spherical array of 57 Meyer Sound MM-4XP loudspeakers around the participant (Fig. 1, left). For the current study, only the front 180° arc of speakers in the central azimuthal plane were used for presenting the stimuli. During the experiment, participants were seated in a chair equipped with a micro PC used to display trial and experiment status information (Fig. 1, right). Participants responded using response buttons mounted to their chair directly to the left and right of the micro PC (Fig. 1, right). The chair and PC were mounted on a mechanical platform that was adjusted to position the participant’s ear level with the speaker array.

### Procedure

Participants performed a change perception task followed by a cued recall task in three blocks of 333 trials (999 total). Both of these tasks are described in detail in the sections that follow. Each block took about 45 minutes to complete and participants were given a brief break between blocks. With informed consent, hearing screening, experimental blocks, and breaks, the entire session took approximately 3 hours to complete. In keeping with common approaches across existing change deafness studies, participants were given the relatively neutral instruction to judge whether a change had occurred across two presented sound scenes.

#### Change perception task

The change perception task was an AX discrimination task consisting of a brief presentation of two scenes with no feedback. A change occurred (addition or removal of a sound source) on 75% of trials; the remaining 25% were catch trials where no change occurred. Presentation of scene A was followed by a 750 ms inter-stimulus interval and then scene X was presented. Following the AX presentation, participants were asked via a screen text prompt “*did a change occur?*”

**Fig. 2:**
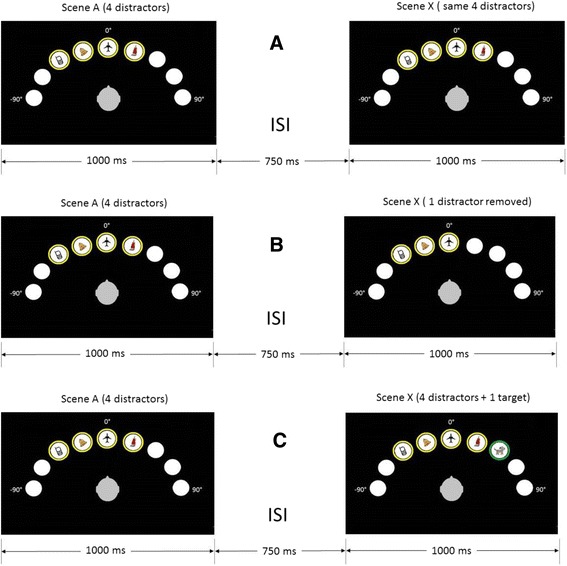
Trial structure was the same for both the spatially separated (multi) and spatially co-located conditions (mono); listeners would hear scene A (1000 ms) and after a brief inter-stimulus interval scene X. The *top* (**a**) panel depicts a No-change trial, the *middle* (**b**) panel a Remove trial, and the *bottom* (**c**) panel an Add trial

Change scenes were constructed by pseudo-randomly selecting five sounds from the full set of 25 sounds described in the Stimuli section, above. Each of these sound arrays was presented for a duration of 1 s. For Add trials, four sounds were presented in scene A and the fifth sound was introduced in scene X. For Remove trials, five sounds were still selected, however, the fifth sound was ‘ignored’, with only four of the sounds being presented in scene A; a single source was selected for removal in scene X. The changed source was considered the target and the remaining sources were considered background or contextual sounds. Figure 2 depicts examples of No-change, Add, and Remove trials. Sampling from the set of 25 sounds was pseudo-random, without replacement. Subsequent trials were constructed using the same process with the remaining 20 sounds, until the set was empty. The process was repeated with successive new sets of the full 25 sounds until each of the targets was represented 15 times in scenes for ‘Add’ trials and 15 times for ‘Remove’ trials, for a total of 750 unique change trials for each participant. No-change trials were created in the exact same manner as change trials except that, in both scene A and X, four sounds were present.

The scenes for the change perception task were presented in two different spatial configuration conditions, a Spatially Separated configuration, where each sound in the scene occupied a unique position in space, emitting from its own, separate speaker, and a Spatially Co-located condition where all of the sounds in the scene were emitted from a single speaker positioned directly in front of the listener. In the multi-speaker condition, scenes were pseudo-randomly mapped to one of three spatial regions across the nine speaker array, namely (1) a central region of speakers straddling 315° to 45°, (2) a leftward region straddling 270° to 0°, and (3) a rightward region straddling 0° to 90°. In all regions, adjacent speakers are separated by 22.5°. Once assigned to a spatial region, the sound elements in a scene were each randomly mapped to one of the five possible positions within a region.

#### Memory recall

Memory for the individual sources presented during the change perception task was probed in a cued recall task following each change and no-change trial. Because the memory task always followed the AX task the performance observed may represent a lower-end estimate of auditory cued-recall performance. The memory recall task was structured such that, on 75% of trials, listeners were presented with a valid probe; that is, the probe was a sound source that was present in both scene A and scene X, and thus was always one of the non-changing background sources, and never the change item itself. On the remaining 25% of trials a lure was presented (a sound that was not part of either scene). Participants were asked to indicate whether they had heard that sound in the given set of scenes. The cued recall task was always presented immediately following the change perception question for two reasons. First, to measure the influence of set size in the change perception task on recall accuracy, there are probe present and probe absent trials for Add (5 sounds), No-change (4 sounds), and Remove (3 sounds) trials. Second, although it is possible that a delay in asking the cued recall question after each AX trial may reduce accuracy, it eliminates the possibility that participants are cued to attend to a particular source prior to answering the AX question (for a similar approach and discussion, see Mitroff et al., 2004).

#### Counting control test

In the change discrimination task, the scenes were constructed such that scene A always contained four sound sources; thus, there was the possibility that participants could complete the AX task by counting sources rather than comparing scenes A and X. To ensure that participants were not simply counting the sounds presented in scene A and comparing the count to the number in scene X, a 150 trial control was conducted to evaluate the ‘counting sounds’ strategy. A single scene containing 3, 4, or 5 sounds (random across trials) was presented on each trial. Eight participants used a keypad to indicate the number of sounds present in the scene. In general, performance on this task was quite poor, with an average proportion-correct of only 0.16. Additionally, there was a significant difference in the proportion-correct based on the number of sounds presented. As the number of sounds increased, accuracy decreased (*M*_*3*_ = 0.32, *M*_*4*_ = 0.12, *M*_*5*_ = 0.05, *F* (2,14) = 10.08, *P* = 0.02), but the main point of note is that performance was poor at every level. This outcome demonstrates that it is difficult for listeners to determine the exact number of sounds present within a relatively short duration auditory scene with three or more individual environmental sound sources.

## Results and Discussion

Tables 2 and 3 show hit and false alarm rates averaged across participants for each of the conditions within the change discrimination and cued recall tasks, respectively. In the change discrimination task, hit rates were calculated based on correct responses on change trials and false alarms are based on incorrect responses on no-change trials. Similarly, in the cued recall task, hit rates were based on correct responses to the probe sound for probe present trials, while false alarms were based on incorrect responses on probe absent trials. In addition to reporting hit and false alarm rates, SDT analyses were applied to estimate listener sensitivities (*d’*) that are theoretically free from response bias. Because the change perception task as implemented here is essentially a same/different task, a ‘differencing model’ (described by Macmillan & Creelman, 2005) was used to calculate *d’*. For the cued recall task, *d’* was calculated using a yes/no model (Macmillan & Creelman, 2005). The results are discussed in the context of both accuracy and sensitivity; however, inferential statistics are performed on *d’* values only.

**Table 2:** Sensitivity (*d’*), hits, and false alarms for the change deafness (AX same/different) task. *d’* calculations are based on a differencing model (Macmillan & Creelman, 2005)

	Add			Remove		
	+3 dB	0 dB	–3 dB	+3 dB	0 dB	–3 dB
Spatially Co-located						
*d’*	2.884	2.803	2.898	2.949	2.993	2.962
Hits	0.423	0.399	0.424	0.438	0.452	0.444
False alarms^a^	0.040	0.040	0.040	0.040	0.040	0.040
Spatially separated						
*d’*	4.026	3.275	2.658	3.852	3.325	2.629
Hits	0.681	0.510	0.374	0.650	0.526	0.372
False alarms^a^	0.030	0.030	0.030	0.030	0.030	0.030

^a^False alarms were calculated based on no change (catch trials). Catch trials do not include a change type (add, remove) or a change level (+3, 0, –3) manipulation, as there is no change to be manipulated. Thus, the False alarm rate is the same across all condition bins

**Table 3:** Sensitivity (*d’*), hits, and false alarms for the cued recall question. Values of *d’* were calculated using a yes/no model (Macmillan & Creelman, 2005)

	Add	Remove
Spatially co-located		
*d’*	1.24	1.43
Hits	0.69	0.77
False alarms	0.26	0.26
Spatially separated		
*d’*	1.02	1.55
Hits	0.65	0.75
False alarms	0.26	0.23

Table 2 2 shows hits and false alarm rates, and overall *d’* values averaged across participants calculated using a differencing model, which is appropriate for same/different comparisons (Macmillan & Creelman, 2005) for the Spatially Co-located and Spatially Separated conditions, as a function of change type. For change trials, hit rates varied widely across conditions (from 0.37 to 0.68), but on average were consistent with previous change deafness reports that used 1 s sound samples. For example, the average hit rate of 0.47 across conditions here is equivalent to the 0.47 hit rate reported by Gregg and Samuel (2008) in their Experiment 1. The average false alarm rate here was somewhat lower than Gregg and Samuel (2008) reported (0.04 vs. 0.10), which resulted in a somewhat higher estimate of average *d’* in the present study than the previous study (2.80 vs. 2.22, respectively). Consistent with others (e.g., Puschmann et al., 2013a, 2013b; McAnally et al., 2010), average *d’* values across conditions were fairly high, indicating that listeners were quite sensitive to changes in the sound scenes.^1^

For the cued recall task, hit rates were high (0.65–0.77); however, false alarm rates were much greater than found in the change discrimination task (Table 3). Estimates of sensitivity during cued recall were calculated using a yes/no model (Macmillan & Creelman, 2005) and resulted in *d’* values ranging from 1.02 to 1.55. These *d’* values correspond to [*p(c)*_*unb*_] values ranging from 0.70 to 0.77 (where [*p(c)*_*unb*_] estimates performance of an optimal unbiased observer), which are very similar to the observed hit rates reported in previous studies (Gregg & Samuel, 2008).

### Change discrimination results

An ANOVA with the within subjects factors of Trial Block Order (1st, 2nd, 3rd), Change Type (Addition, Removal), and Change Level (–3, 0, +3 dB), and the between subjects factor of Spatial Separation (Co-located and Spatially Separated), was performed on the calculated *d’* values. Post hoc testing was performed using Tukey’s Honestly Significant Difference test. First, the main effect of change type was not significant (*F* < 1). The main effect of Block Order was only marginally significant (*F* (2, 48) = 3.05, *P* = 0.057, _*p*_ *η* ^*2*^ = 0.11). There was a small gradual decline in sensitivity across blocks; however, none of the post-hoc pairwise differences were significant (*M*_*diffs*_ < 0.2, *P* > 0.05).

**Fig. 3:**
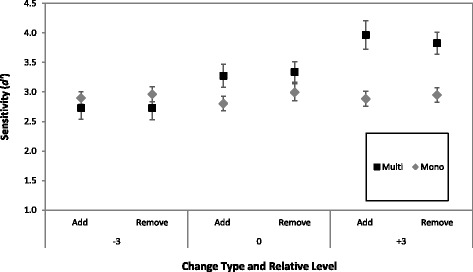
Sensitivity in the change discrimination task as a function of spatial condition, change type, and relative target level, with SE *bars* shown

The main effect of Change Level (–3, 0, +3 dB) was significant with a large effect size (*F*(2, 48) = 107.05, *P* < .0001, _*p*_ *η* ^*2*^ = 0.82); errors systematically increased as relative level decreased, with each level significantly different from the lower one (*M*_*diff*_ > 0.27, *P* < 0.05; Fig. 3). This random application of a level difference irrespective of change type was introduced to avoid participants basing their responses simply on possible perceived changes in loudness due to the addition or subtraction of a sound source. The observed monotonic relationship between change level and change discrimination performance was not surprising, given the differences in target-background saliency that resulted from the combination of change type and change level.

Including all three target-background levels, the main effect of the between-subjects manipulation, Spatial Separation, was not significant (*F* (1, 24) = 3.23, *P* = 0.085, _*p*_ *η* ^*2*^ = 0.12). However, there was a significant interaction and large effect size between Change Level and Spatial Separation (*F* (2, 48) = 112.14, *P* < 0.0001, _*p*_ *η* ^*2*^ = 0.82). Figure 3 clearly shows that the interaction is due to an essentially flat function of performance and relative target-background level in the Spatially Co-Located scenes condition, while change discrimination is a strong monotonic function of target-background level in the Spatially Separated scenes condition. Post-hoc tests revealed that, in the Spatially Separated scenes condition, the –3 dB relative target level produced the worst performance, which is somewhat expected given the low target-to-background signal-to-noise ratio. This condition (–3 dB, with spatial separation) was the primary contributor in washing out the effect of spatial separation in the overall ANOVA. By excluding the –3 dB level data in the Spatially Separated sources condition, a convincing pattern emerges; an advantage of spatial separation on change discrimination, with sensitivity in both the 0 and +3 dB levels significantly greater than any of the three relative target levels in the Spatially Co-located condition (*M*_*diff*_ > 0.37, *P* < 0.05). In terms of accuracy (also excluding data from the –3 dB level), the smallest difference in hit rate between equivalent target background levels was more than 0.07 for the 0 dB level and more than 0.17 for the +3 dB level.

A secondary question was whether the relative spatial region of the sound scenes affected change discrimination. In localization, typically, spatial precision is best directly in front of the listener and worse at lateral positions (e.g., Middlebrooks & Green, 1991). To address this question, a follow-up repeated-measures ANOVA was performed on the data from the Spatially Separated sources, and the results showed that the effect of spatial region was not significant (*F* (2, 24) = 2.01, *P* = 0.15, _*p*_ *η* ^*2*^ = 0.15). The lack of significance for spatial region may be due to the spacing of the loudspeakers at intervals of 22.5°, which is much greater than localization precision for broadband sounds presented in isolation (Middlebrooks & Green, 1991).

### Memory recall results

A repeated-measures ANOVA with the within subjects variables of Trial Block Order (1st, 2nd, 3rd), Change Type (Add, Remove, and None) and the between subjects variable of Spatial Separation (Co-Located and Spatially Separated) was performed on the calculated *d’* values. The main effect of trial block order was not significant (*F* < 1) and neither was the main effect of Spatial Separation (*F* < 1). Change type, however, was significant (*F* (2, 48) = 10.79, *P* < 0.0001, *η* ^*2*^ = 0.31), with Remove trials sensitivity (*d’* = 1.49, SE = 0.07) higher than that of No-change trials (*d’* = 1.29, SE = 0.08) and Add trials (*d’* = 1.13, SE = 0.06). The two-way interaction between change type and spatial condition was not significant (*F* (2, 48) = 2.58, *P* = 0.09, _*p*_ *η* ^*2*^ = 0.10) and neither were the interactions between block and change type, or block and spatial condition (*F* < 1). However, the three-way interaction between change type, block order, and spatial separation was significant (*F* (4, 96) = 3.01, *P* < 0.02, _p_ *η* ^*2*^ = 0.11).

**Fig. 4:**
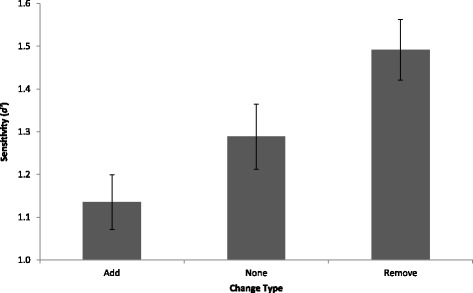
Sensitivity in the cued recall task as a function of change type in the change discrimination task with SE *bars* shown

These results, which are shown in Fig. 4, indicate that listener sensitivity systematically decreased as the number of sounds in the second scene increased. When a source was added in the change deafness task, the number of sounds increased from 4 to 5. For No-change trials, the number of sounds remained constant at 4, and in Remove trials, the number of sounds went from 4 to 3. This pattern was also reflected in corresponding hit rates with Remove trials producing the highest hit rates ([*p(c)*] = 0.76, SE = 0.01), followed by No-change trials ([*p(c)*] = 0.68, SE = 0.02) and Add trials ([*p(c)*] = 0.67, SE = 0.02). This result makes sense from the perspective that an increase in the number of items should increase memory demands and, more specifically, should affect the ability to recall all the sounds presented within the span of such a short (1 s) interval.

## General discussion

These results suggest that, while the type of change introduced did not influence errors, a host of other common factors did have an impact. The amplitude of the change relative to the background influenced performance and providing spatial separation among the individual sources in the scene improved change perception. Performance on the memory task was somewhat better than on the change discrimination task and, contrary to the change discrimination task, the analysis of recall performance showed a significant effect of change type (Add, Remove, No-change). These results suggest a change in memory load as a function of change type; differences in scene size may have impacted change perception errors due to changes in the memorability of scenes of difference sizes. Each of these main findings will be discussed further in the sections that follow.

### The influence of spatial separation

The current study found a significant beneficial effect of spatial separation on change errors. This effect was strongly modulated by change level. Indeed, when the relative target level was equal to the average background level, there was a 7% decrease in errors in the spatial versus co-located conditions, and the decrease in errors was as large as 17% when the change target was 3 dB above the average background level. As in the current study, spatial separation using real loudspeaker locations gives the advantage of multiple spatial segregation cues for these broadband environmental sounds that include binaural phase, amplitude and monaural spectral cues that reflect transfer functions related to individual physiology and room effects (see Middlebrooks & Green, 1991 for a review). This spatial advantage is somewhat larger, but also consistent with previous reports by Eramudogolla et al. (2005), who implemented individualized head-related transfer functions presented over headphones. We speculate that the failure of previous reports to find an advantage for spatial segregation may have been due to artificially restricting spatial cues to a single dimension such as amplitude panning (e.g., inter-aural level; Gregg & Samuel, 2008), rather than presenting signals with multiple spatial cues such as in the present study.

### The link between memory and scene size

The change type manipulation was not significant in the change discrimination task, suggesting that the manner in which a change is induced does not affect the perception of that change. In the current study, the change type manipulation affected overall scene set size. This did not matter for measures of change perception, but was meaningful in the measure of recall ability. The monotonic relationship between scene size and both hit rate and sensitivity suggests evidence of a capacity limit for simultaneously occurring sources. It is possible that, in the 1 s exposure to each scene, listeners were not able to effectively encode each of the individual sound sources. This assertion is based on the set size effect observed in the data presented here, but also the set size effect reported by others (Gregg & Samuel, 2008; Eramudugolla et al., 2005; McAnally et al., 2010). This notion is also consistent with McAnally et al.’s (2010) finding of reduced errors as scene durations increased from 1 to 3 to 5 s. Presumably, the increases result in better object encoding and thus a reduction in change deafness errors.

### The benefit of sensitivity and accuracy as measures of change perception performance

In the present study, hits and false alarms were used to calculate sensitivity (*d’*) for both the change discrimination and the cued recall task. Sensitivity has been measured in other studies of change deafness (e.g., Eramudugolla et al., 2005; McAnally et al., 2010; Puschmann et al., 2013b), but reported inferential statistics have often been based on accuracy rather than *d’* (see Gregg & Samuel, 2008, for discussion of exclusion of *d’* analysis). Consistent with others, the present study finds that listeners are generally quite poor at noticing a change when it does occur (low hits and high misses), but perform well on No-change trials (low false alarms and high correct rejections), indicating that listeners are nearly perfect at judging when no change has occurred. This pattern is indicative of a conservative response criterion; participants only respond ‘yes’ when they are quite certain that a change has occurred. Mitroff et al. (2004) note that this response pattern is a common occurrence for participants in visual change detection studies. Indeed, they gave specific instructions to promote a more liberal response criteria to offset the ‘default’ participant conservative criteria. The real benefit of SDT analyses is that they allow an unbiased evaluation of change deafness manipulations. In the current study, our results demonstrate that, despite a conservative response criterion, measures of sensitivity show evidence of change errors (and thus, change deafness) that are also reflected in the relatively high miss rates.^1^

### Similarity relationships between scene elements

In a recent review, Dickerson and Gaston (2014) suggested that factors, such as perceptual similarity among simultaneously presented sources, might play a strong role in listening tasks involving complex sound events. This notion is also consistent with Gregg and Samuel (2009), who found that manipulation of simple acoustic properties or sematic similarity could significantly influence change errors; specifically, reducing similarity reduces the likelihood of errors. One of the novelties and strengths in the present study was the large number of combinations of the sound to be changed (i.e., added or removed) and background sounds. Other studies of change deafness incorporated relatively large sets of representative environmental sounds (see Eramudugolla et al., 2005 and Gregg & Samuel, 2008, for examples), but included only small subsets of possible change-to-background combinations. In addition, often the specific change-to-background relationships were left undefined or were only partially characterized. In the present study, each of the 25 targets was presented 15 times and distractor sounds were pseudorandomly assigned on each trial for each listener. This mapping resulted in an incredible amount of variability in change target/distractor scene combinations, with many presentations of each possible combination occurring across participants in the almost 26,000 total trials (75% change and 25% no-change trials, 999 trials per participant). The large amount of data provided a unique opportunity to further examine the link between sound source similarity and change perception performance. To accomplish this goal we conducted two follow-up analyses.

**Fig. 5:**
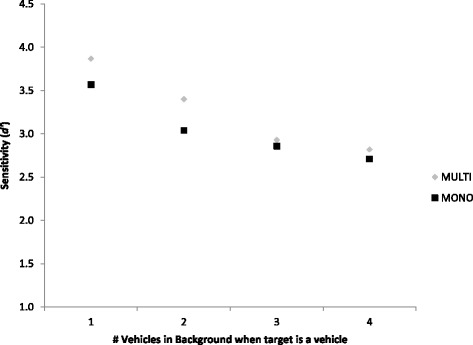
Sensitivity in the change discrimination task as a function of auditory scene similarity. When the data were re-analyzed based on vehicle category membership, a significant reduction in sensitivity was observed as category-level similarity increased

**Fig. 6:**
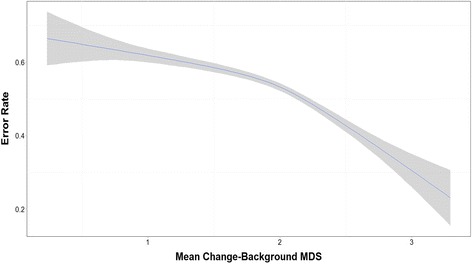
*Trend line* with bounds (standard error) showing the relationship between the average similarity (in multidimensional scaling space) between change targets and background sounds as a function of error rate in the change discrimination task

The first analysis looked at simple, category-level similarity by sorting all trials to create a bin where the change item was always a member of a single category; in this case, a vehicle sound. These trials were then sorted into bins where at least one and up to four of the background sounds was also a vehicle sound. Figure 5 plots the average *d’* values for each of these bins and shows a clear monotonic relationship between category-level similarity and change deafness sensitivity. In the second analysis (aspects of this analysis and specific details can be found in Dickerson et al., 2016),^3^ trial-wise change deafness data, represented as a binary correct or incorrect score, was plotted against a composite similarity score. Composite similarity was calculated by collecting similarity data in a pilot study where listeners rated pairs of sounds from the 25 experimental sounds for their overall similarity. These data were then analyzed using multidimensional scaling (MDS), and generated a two-dimensional MDS solution. The composite similarity score was calculated for each unique trial by taking the average Euclidean distance in MDS space between a given change and each of the background sounds presented within a particular scene. Figure 6 shows the relationship between the composite similarity score and change errors. From this and the category-level vehicle relationship, it appears that the similarity between a change and the background is predictive of change perception errors or ‘change deafness’ in complex scenes. This conclusion is consistent with previous work in our lab showing a link between perceptual similarity and discrimination performance (Gaston & Letowski, 2012). However, this conclusion is based on post hoc analyses, and thus must remain tentative until these relationships are directly manipulated. This is the focus of an ongoing set of experiments in our lab.

## Conclusion

The present study integrates many of the previous change deafness manipulations and contributes meaningfully to a small but growing number of voices suggesting that change deafness is a distinct perceptual phenomenon represented by a particular pattern of performance, namely low hits and low false alarms. However, despite similar nomenclature, the temptation to draw links between change deafness and its visual counterpart (change blindness) should be avoided. There are methodological and physiological differences between vision and audition that would have to be ignored to make direct comparisons between the two effects tenable. For example, the AX paradigm and accuracy measures that are standard in the change deafness literature are essentially a single ‘flicker’ and thus are only roughly analogous to single flicker or ‘one-shot’ change discrimination paradigms in vision (e.g., Mitroff et al., 2004). In standard flicker paradigms, multiple flickers can occur, and the primary dependent measure is reaction time to identify the change. When the change is found, the dependent measure of accuracy is almost always asymptotic. In contrast, the majority of change deafness studies are based on only one ‘flicker’ and thus the dependent measure is restricted to accuracy following a restricted exposure window that limits the effective time to encode scene elements. The two methods are fundamentally different, and thus difficult to compare. One possible solution is to provide analogous situations by designing auditory flicker paradigms where the goal is instead to identify the auditory change, and the dependent measure is response time to identify the change (e.g., Hall et al., 2015).

## Footnotes

1 This paper includes both *d’* and accuracy analyses in order to gain a better understanding of the relationship between sensitivity and accuracy in reporting the presence of a change. We acknowledge that change deafness is defined as missing a change that is presented, and not reporting a change that has not been presented and thus *d’* is a measure that does not capture change deafness directly. However, by reporting both measures it is possible to develop a view of change deafness that takes into account the role of bias and uncertainty, as well as estimate overall sensitivity. Further, while *d’* can capture perceptual sensitivity to information it does not necessary represent the higher-level cognitive processes that would influence errors reporting changes in the type of complex stimuli presented in change deafness tasks.

2 These responses are based on a ratio of 75% change and 25% no-change trials. This was selected to maximize the number of change trials collected for each IV (independent variable), while providing a reasonable estimate of no-change performance. Given the task with no feedback, there is little reason to assume that participants would learn the trial distribution. Thus, in the following analyses, no correction is applied to averaged data for the differences in the trial distribution for change and no-change trials. Average responses for each response type are weighted equally.

3 This preliminary pilot data was presented as part of a poster session at the Spring 2015 Meeting of The Acoustical Society of America and was recently published in the Proceedings of Meetings on Acoustics.
